# Food Security Resilience and Humanitarian Aid in Mali: A Case Study of Bandiagara Cercle

**DOI:** 10.1155/ijfo/2415147

**Published:** 2024-12-04

**Authors:** Innocent Mumararungu, Gisaro Ca-Madeberi Ya-Bititi, John Rwirahira, Philippe Burny

**Affiliations:** ^1^Laboratory of Economics and Rural Development, Gembloux Agro-Bio Tech, University of Liège, Gembloux, Belgium; ^2^College of Arts and Social Sciences (CASS), University of Rwanda, Kigali, Rwanda

**Keywords:** climate change, food security, humanitarian aid, Mali, resilience, socioeconomic challenges

## Abstract

Mali's food security strategies focus on improving agriculture, water management, and diversifying livelihoods. While initiatives like climate-smart agriculture show promise, challenges like limited resources, market access, and political instability persist. Gender inequalities and reliance on external aid further hinder progress, making it difficult for Mali to build sustainable, self-reliant food systems and ensure long-term resilience. This research assessed the effectiveness of humanitarian aid interventions in enhancing food security resilience in the Bandiagara Region of Mali. It is aimed at evaluating the quality and sufficiency of the food provided to beneficiaries, as well as the alignment of aid efforts with local needs. The study employed a mixed-methods approach, combining quantitative household surveys with 295 respondents across 14 villages and qualitative data through semistructured interviews with 37 local authorities selected on purposive sampling and focus group discussions with beneficiaries. The findings indicated that while the aid interventions were generally relevant and well executed, with beneficiaries expressing satisfaction with the quality of the millet provided, there were significant concerns regarding the quantity of food distributed. Many beneficiaries felt that the portions were insufficient to meet their needs, especially in the context of recurring food shortages. This highlighted the need for more tailored, context-specific aid allocation strategies, ensuring that the quantity of food provided aligns better with local requirements and the scale of food insecurity, thereby enhancing the overall effectiveness of humanitarian support. It is recommended to expand and continue millet distribution programs, with enhanced monitoring mechanisms to ensure resource effectiveness. Emphasizing customized food allocation and increased community engagement will strengthen local ownership and resilience. By aligning aid with local needs and improving intervention targeting, these strategies are aimed at creating a more sustainable and equitable food security system in the Bandiagara Region, better equipping it to withstand future food insecurity challenges.

## 1. Introduction

Food security remains a persistent global challenge affecting both developed and developing nations, with its complexity intensified by factors such as population growth, changing dietary preferences, and the effects of climate change [1]. Defined by the 1996 World Food Summit, food security occurs when all people have consistent access to adequate, safe, and nutritious food necessary for a healthy life [2]. This concept extends beyond mere availability to include accessibility and stability, evolving since the 1970s to incorporate the four dimensions of availability, access, utilization, and stability, thus acknowledging individual vulnerability. According to the FAO, food security exists when people consistently have physical, social, and economic access to sufficient, safe, and nutritious food aligned with dietary preferences for an active, healthy life [3]. In contrast, food insecurity—defined at the household level by the US Department of Agriculture (USDA)—reflects limited or uncertain access to adequate food, distinct from hunger, which is a physiological condition that may arise from food insecurity [4]. Key factors contributing to food insecurity include high housing costs, social marginalization, economic discrimination, chronic health issues, medical expenses, and low income, which collectively impact quality of life and exacerbate food insecurity among vulnerable populations.

The 2030 Sustainable Development Goals (SDGs) set by the United Nations highlight food security as a central issue, linking it with sustainable agriculture. Specifically, SDG 2 is aimed at ending hunger, ensuring food security, enhancing nutrition, and promoting sustainable agriculture, with interconnected targets underscoring the relationship between food security, nutrition, and agricultural sustainability [5]. Global leaders are committed to eradicating poverty in all forms, including extreme poverty, through social protection systems, emphasizing rural development and sustainable agriculture. Initiatives like the Rome Declaration on Nutrition and the Framework for Action advocate for a comprehensive global food system that addresses nutritional needs while conserving environmental resources, underscoring the role of the Committee on World Food Security.

Achieving food security universally requires increasing food production while improving accessibility, affordability, and food utilization. Sustainable agricultural practices—such as agroecology, conservation agriculture, and crop diversification—are pivotal in enhancing resilience against climate change, reducing resource depletion, and promoting productivity [6]. Despite these efforts, eradicating food insecurity by 2030 remains a formidable challenge. Recent data estimates that between 691 million and 783 million people globally experienced hunger in 2022, marking an increase of 122 million from prepandemic levels in 2019 [7]. Africa, in particular, faces a high incidence of hunger, with nearly 20% of the population affected compared to 8.5% in Asia and 6.5% in Latin America and the Caribbean. Projections suggest that nearly 600 million people may experience chronic undernourishment by 2030, posing a significant barrier to achieving SDG 2. Furthermore, urban areas report lower food insecurity rates compared to rural and periurban regions, where 33.3% of adults in rural areas experienced moderate or severe food insecurity in 2022, in contrast to 28.8% in periurban and 26.0% in urban settings [7]. These disparities emphasize the critical need for resilience building in food security efforts.

**Figure 1:**
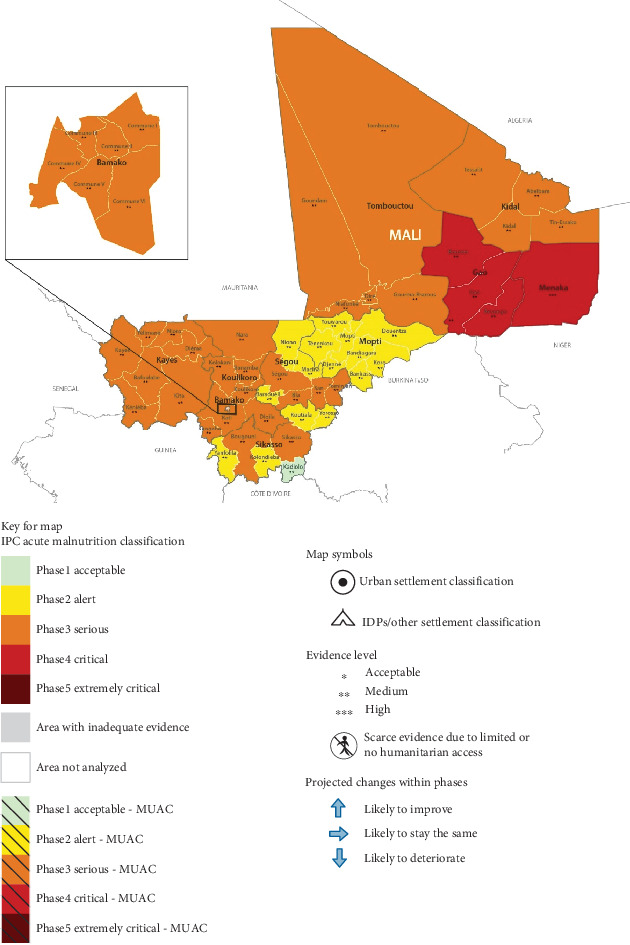
Map of projected malnutrition in Mali from November 2023 to May 2024. *Source:* [10].

In the Sahel, food security challenges are intensified by fragile ecosystems and climate variability, particularly in regions where agriculture and livestock heavily depend on rainfall. Low rainfall, especially between March and June, limits access to pasture and water, exacerbating food insecurity for Sahelian communities during droughts [8]. Mali, a landlocked Sahelian nation, faces these climate challenges alongside political instability and external pressures. In 2023, despite economic resilience, Mali's food security continued to be strained, with over 715,000 people experiencing acute food shortages a figure expected to nearly double to 1.4 million in the 2024 lean season, with a significant increase in areas classified as “crisis phase” (Integrated Food Security Phase Classification (IPC)-3) [9] as indicated by IPC in Figure 1.

The implications of food insecurity for Mali's young population are severe, with an estimated 1.4 million children under five facing acute malnutrition from June 2023 to May 2024. This includes 313,185 severe acute malnutrition (SAM) cases and 1.1 million moderate acute malnutrition (MAM) cases, along with 87,865 acutely malnourished pregnant or lactating women in need of treatment. Contributing factors include poor dietary intake, food insecurity, and common childhood illnesses like diarrhea, respiratory infections, malaria, and measles. Additionally, suboptimal breastfeeding practices exacerbate malnutrition [10]. Since 2017, Mali has been implementing its National Food and Nutritional Security Policy (PolSAN) to improve food security and nutrition, particularly for vulnerable groups, aligning with its Strategic Framework for Economic Recovery and Sustainable Development (CREDD) [11].

Despite these policies, food insecurity remains a pressing issue in Mali, a Sahelian country where 68% of the workforce is engaged in agriculture, livestock, fishing, and forestry sectors vulnerable to recurrent droughts and floods [12]. The 2022 inflation in global fuel and food prices, exacerbated by the Russia–Ukraine conflict, has further strained Mali's food security [13], compounded by poor agricultural output and insecurity despite a projected increase in cereal production for the 2022–2023 season [14]. In response, Caritas Luxembourg and Caritas Switzerland launched an emergency project in Bandiagara to address urgent food and nutrition needs, aiming to enhance resilience to food insecurity.

Building resilience is essential for enabling communities to withstand food insecurity challenges in the Sahel. Resilience, defined as the capacity of a system to absorb shocks while maintaining function, is crucial in fragile regions [15]. However, fostering resilience in contexts like the Sahel requires holistic approaches addressing socioeconomic and environmental challenges, as fragile livelihoods and diminishing resources exacerbate food insecurity [16]. Integrating resilience-building strategies into policy frameworks is essential to tackling the root causes of instability and promoting sustainable development in the Sahel. A comprehensive approach, combining humanitarian assistance with resilience building, is necessary to enhance food security and community well-being. By synthesizing insights from ecology, engineering, and economics, policymakers can formulate strategies to address food insecurity drivers and foster sustainable development in vulnerable regions like the Sahel [17–19]. Since 2012, Mali's security crisis—characterized by armed group conflicts and intercommunity tensions—has disrupted socioeconomic activities, displacing populations and undermining food security. Bandiagara Cercle in Mopti is especially affected by security and climate challenges, including flooding and heavy rains, which threaten agricultural production and food access for the most vulnerable [14].

## 2. Problem Statement

While humanitarian aid plays a vital role in alleviating immediate food insecurity, there is a significant gap in research exploring its long-term effectiveness in building resilience against future shocks, particularly in fragile regions like Bandiagara. Understanding how humanitarian interventions, such as food distributions, contribute to lasting resilience in the face of climate change, political instability, and socioeconomic vulnerability remains underexplored. There is a lack of in-depth analysis regarding how well humanitarian aid interventions align with the specific needs of local communities. While food security programs often focus on immediate relief, their design may not fully account for the unique cultural, environmental, and socioeconomic contexts of the populations they aim to assist. Research exploring how aid is tailored to local realities, including food preferences and agricultural practices, would improve the effectiveness of aid delivery.

This study advances current food security research by examining the intersection of humanitarian aid, climate resilience, and socioeconomic factors in Mali's Bandiagara Cercle—a region acutely affected by food insecurity. Unlike broad analyses, this study provides a focused examination of local challenges in a Sahelian region marked by climate volatility and sociopolitical instability. By assessing the adequacy of food distributions (particularly millet) and beneficiary satisfaction, the study contributes a nuanced understanding of aid effectiveness in fragile environments, addressing gaps in resilience-focused humanitarian interventions. The research highlights the impact of culturally relevant food distributions on resilience and food security, underscoring the need for humanitarian interventions that extend beyond immediate food provision to integrate climate resilience and community-specific needs into long-term strategies. This study's findings align with SDG 2 and have implications for designing evidence-based adaptations in aid programs to achieve sustainable food security in vulnerable regions. The research is aimed at exploring key aspects of food security resilience and the role of humanitarian aid in the Bandiagara Cercle of Mali. Specifically, the study seeks to assess the alignment of humanitarian aid with the immediate food security needs and priorities of beneficiaries, evaluating the effectiveness of its utilization in addressing food security challenges within targeted communities. Additionally, the study is aimed at analyzing both the quality and quantity of the distributed aid and its impact on improving food availability, accessibility, and consumption patterns among vulnerable households. Furthermore, the research will investigate how humanitarian aid initiatives contribute to long-term food security resilience by addressing underlying vulnerabilities, fostering community participation, and promoting sustainable practices to ensure lasting improvements in food security.

## 3. Methods

**Figure 2:**
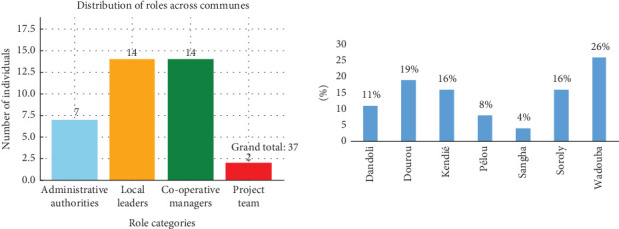
Number of selected respondents in purposive sampling and percentage of respondents/households by commune (field data December 2023).

The study targeted 1286 households, all recipients of humanitarian assistance, across seven communes in Bandiagara. A mixed-methods approach was employed, combining purposive and simple random sampling techniques to achieve both comprehensive coverage and representative sampling. Purposive sampling was used to select 37 respondents from specific groups critical to the study, including administrative authorities (mayors and municipal councilors), local leaders (village chiefs), and leaders of cooperatives or agricultural professional organizations (OPA), as illustrated in Figure 2. Simple random sampling was applied to select household respondents across the seven communes. The number of households surveyed per commune was proportional to the percentage of households that received assistance in each commune. Rather than using control groups of nonbeneficiaries, the study compared beneficiaries across the communes. This decision was guided by ethical considerations, particularly the humanitarian principles of “do no harm” and the commitment to providing equitable and impartial assistance. Including nonbeneficiaries as a control group could have raised significant ethical concerns, such as withholding essential aid, and posed practical challenges, including difficulties in securing informed consent and cooperation from excluded individuals. By focusing exclusively on communes where all participants received aid, the study avoided these issues. Furthermore, this approach allowed for an evaluation of how local contextual factors such as infrastructure quality, access to services, and socioeconomic conditions affected the effectiveness and reception of humanitarian assistance. With a 95% confidence interval and a 5% margin of error, a sample size of 295 households was determined for interviewing, ensuring the reliability and validity of the study's findings, as illustrated in formulas below:

 $$\begin{array}{c} n=\frac{N\cdot z^{2}\cdot p\cdot \left(1-p\right)}{\left(E^{2}\cdot \left(N-1\right)+Z^{2}\cdot p\cdot \left(1-p\right)\right.} \end{array}$$

where *N* is the total population size (1286 households), *Z* is the *Z*-value corresponding to the desired confidence level (for 95%, *Z* = 1.96), *p* is the estimated proportion of the population (for conservative estimation, *p* = 0.5 which maximizes sample size), and *E* is the desired margin of error (0.05 for 5%).

Therefore,

 $$\begin{array}{c} n=\frac{1286*\left(1.96\right)2\;\left(0.5\right)*\left(1-0.5\right)}{\left(\;0.05\right)2*\left(1286-1\right)+\left(1.96\right)2*\left(0.5\right)*\left(1-0.5\right)} \end{array}$$

 $$\begin{array}{c} n=\frac{1286*3.8416*0.25}{0.0025*1285+3.8416*0.25}n=\frac{1286*0.9604}{3.2125+0.9604}n=\frac{1235.76}{4.1729}=295.01 \end{array}$$

The household survey data indicates a clear gender disparity, with men predominantly responding to the questionnaires (64%) and men also leading the majority of surveyed households (68%). This suggests that men typically hold the primary decision-making role in households, reflecting a patriarchal structure. Meanwhile, a smaller proportion of women (36%) were respondents, with 28% identified as spouses of the male household heads. The limited representation of children and parents (both at 2%) in the survey highlights that household leadership and responses are concentrated among the heads and their spouses, potentially underrepresenting the perspectives of younger and older generations.

### 3.1. Ethic Consideration

Ethical considerations included obtaining community permission; ensuring participant anonymity and confidentiality; securing informed consent; and respecting local languages, cultural practices, and religious beliefs. The study emphasized voluntary participation, data security, and inclusivity, particularly for vulnerable groups, while refraining from offering any form of compensation to respondents.

### 3.2. Limitation

The fieldwork faced limitations due to the harvest season affecting beneficiary availability, increased reluctance to participate amid rising insecurity in the Bandiagara area, and difficulties in meeting with the mayor of the Sangha Commune due to local security concerns, though the data collection objectives were ultimately met.

## 4. Results and Discussion

### 4.1. Main Source of Household Income

**Figure 3:**
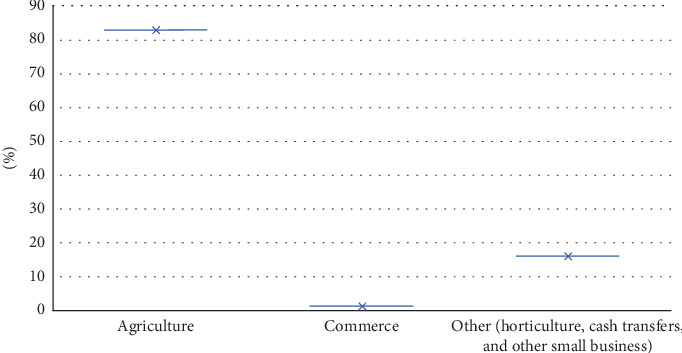
Source of main income of respondents (field data December 2023).

The findings demonstrate in Figure 3 a critical reliance on agriculture, with 83% of households depending on it as their primary livelihood, highlighting significant vulnerability to agricultural risks such as climate change, pests, and market fluctuations. The limited diversification of income sources—16% engaging in other activities such as horticulture, cash transfers, and other small business—indicates a fragile economic base, with minimal resilience to shocks. The negligible participation in commerce (1%) reflects an underdeveloped market economy, constraining broader economic growth and household adaptive capacity. From a food security perspective, this dependence on agriculture underscores the need for humanitarian assistance in the lean season to enhance agricultural resilience, promote income diversification, and invest in market development to build economic stability and reduce vulnerability.

### 4.2. Relevance and Utilization of Humanitarian Aid

In regions like Mali, characterized by recurrent food insecurity exacerbated by climate-related and socioeconomic challenges, humanitarian aid interventions play a pivotal role in mitigating the impacts of crises [20]. However, the effectiveness of such interventions is often hindered by their delayed arrival, typically occurring well into the lean season when communities have already been enduring months of hardship [21]. This delayed response underscores the critical need for timely and proactive measures to address food insecurity, particularly in vulnerable regions like Mali. The distribution of millet was generally tailored to the context of an increasingly thorough understanding of the community's needs. Indeed, the implementing partner in the Bandiagara Cercle, Caritas Switzerland, conducted a needs assessment based on food deficits linked to the drought caused by low rainfall in 2021 and partial flooding due to heavy rainfall in 2022 in Bandiagara. This assessment revealed that 1287 households (11,583 individuals) were at risk of severe food insecurity, leading to food support in the form of cereals at a rate of 0.60 kg of millet per person per day. Furthermore, the assessment indicated that the distribution of millet helped address the priority needs of households during the lean season.

Findings from both household served population and key informant interviews indicate that the distribution of millet effectively addressed the priority needs of households during the lean season, with 100% of respondents across all communes affirming its adequacy, except in Soroly, where 2% expressed dissatisfaction. These respondents attributed their concerns to household compositions exceeding the predefined number of members, including extended family such as cousins, grandparents, nephews, and nieces, which was not considered during distribution. When asked about the alignment of project activities with the population's needs and context, administrative authorities, local leaders, and cooperative representatives overwhelmingly agreed that the project met the population's expressed demands. A cooperative leader from Dounbogou, in the commune of Dandoli, stated, “Yes, it was indeed appropriate because the population's needs were taken into account.” Similarly, a village chief from Soroly remarked, “The assistance came at the right time and was well-suited to the population's needs.” These findings highlight the crucial role of timely and contextually tailored humanitarian assistance in strengthening food security resilience during critical periods such as the lean season. The 100% satisfaction rate, barring a small discrepancy in Soroly, indicates the effectiveness of millet distribution in mitigating immediate food insecurity. However, the challenges in Soroly underscore the importance of adapting distribution criteria to accommodate diverse household structures to ensure equitable coverage. The alignment of project activities with expressed community needs, as affirmed by local stakeholders, reflects the significance of participatory planning and context-specific interventions in humanitarian assistance. This approach enhances the relevance and acceptance of aid, contributing to food security resilience by addressing both immediate needs and the structural vulnerabilities of affected populations.

**Figure 4:**
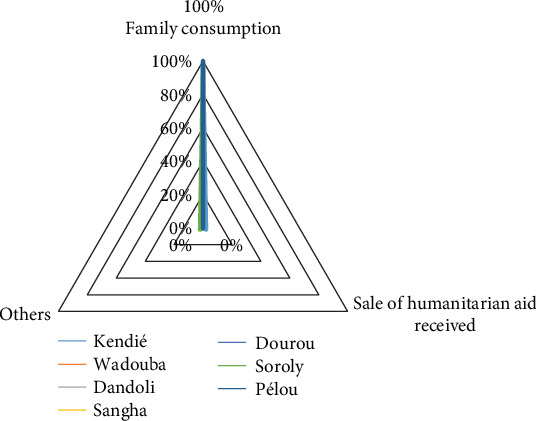
Use of humanitarian aid by commune (field data December 2023).

The data presented in Figure 4 highlights the utilization patterns of humanitarian aid across seven communes in Bandiagara. In most communes, including Dandoli, Dourou, Pléou, Sangha, and Wadouba, 100% of the aid was allocated to household consumption, underscoring a uniform focus on meeting basic needs. However, the communes of Kendié and Soroly display distinct variations. In Kendié, 2% of the aid was sold by recipient families, indicating a small but notable level of economic activity associated with humanitarian assistance. Similarly, in Soroly, while 98% of aid was allocated to family consumption, 2% was diverted to unspecified “other activities,” reflecting a unique utilization pattern in this commune. These findings suggest the need for tailored humanitarian aid strategies that consider the distinct socioeconomic dynamics of each commune. The high allocation to household consumption across most communes indicates a critical reliance on aid to address immediate food security needs, highlighting the vulnerability of these populations to external shocks. In Kendié, the observed economic activity could be leveraged through complementary livelihood programs aimed at income generation and market engagement. Meanwhile, in Soroly, further investigation into “other activities” could inform targeted interventions to address unmet or emerging needs. Effective aid deployment requires a nuanced understanding of local contexts to enhance food security resilience and foster long-term community stability.

### 4.3. Quality and Quantity of Humanitarian Aid

The research emphasized the significance of ensuring the quality and quantity of food distributed through humanitarian aid programs to adequately address nutritional needs and alleviate food shortages.

#### 4.3.1. Quality of Food Distributed

**Figure 5:**
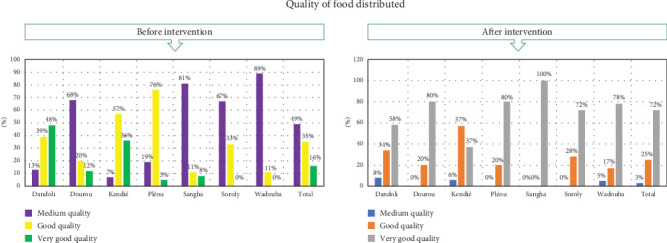
Respondents' opinions on the quality of food received (field data December 2023).

Understanding the level of satisfaction with the quality of millet received by households is a crucial aspect of this research. It is essential to recognize that it is not solely the distribution process that holds significance but also the quality of the food distributed and the degree of appreciation from beneficiaries regarding this quality. Hence, we sought to gauge beneficiaries' perceptions of the quality of millet they received, as outlined in Figure 5.

The research highlights a predominantly positive perception of the millet distributed as humanitarian aid across several communes in Mali. In Dandoli and Pélou, satisfaction levels were particularly high, with 76% and 80% of respondents, respectively, rating the millet quality as *very good* and the remainder as *good*. Similar unanimous satisfaction was observed in Dourou, Sangha, and Wadouba, where 100% of respondents rated the millet as *very good*. In Soroly, while slightly lower at 72% rating the millet as *very good*, the remaining 28% still described it as *good*, confirming widespread acceptance. These findings reflect the success of the millet distribution program in meeting the quality expectations of beneficiaries and addressing food security needs effectively. The data also reveal some variation in perceptions, particularly in Kendié, where 36% of respondents rated the millet as *very good*, 57.5% as *good*, and 3.4% as *average*. While no respondents across any commune rated the millet quality below *average*, the higher proportion of “good” ratings and the small percentage considering it *average* in Kendié suggest a need for further investigation into local preferences or contextual factors affecting satisfaction. These minor discrepancies highlight the importance of tailoring humanitarian aid to account for communal differences to ensure equitable satisfaction and sustained impact across diverse settings. The overwhelmingly positive perception of millet quality underlines the importance of maintaining high-quality standards in food distribution programs to ensure the effectiveness of humanitarian assistance. The program's ability to meet quality expectations strengthens food security resilience by fostering trust and meeting the immediate dietary needs of vulnerable communities. Addressing the observed variations, such as those in Kendié, through participatory feedback mechanisms and continuous quality improvement can enhance the overall impact of such initiatives. By prioritizing quality control, addressing minor perception gaps, and fostering community engagement, humanitarian aid programs can better support long-term resilience and stability in affected populations.

#### 4.3.2. Quantity of Food Distributed

**Figure 6:**
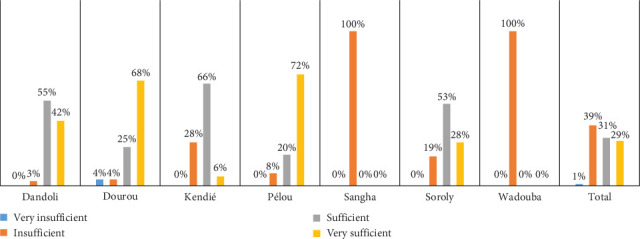
Respondents' opinions on the quantity of millet received (field data December 2023).

The data presented in Figure 6 reveal significant variability in household perceptions of the quantity of millet distributed across different communes in Mali. While 39% of respondents overall found the amount insufficient, 31% considered it sufficient, and 29% rated it as very sufficient. Notably, all households in Sangha and Wadouba reported that the quantity was insufficient, underscoring a uniform dissatisfaction in these areas. This contrasts with households in other communes, where perceptions were more evenly distributed, reflecting mixed satisfaction levels. These differences highlight the complexities of meeting diverse needs across varying contexts within the same humanitarian aid program.

Despite the dissatisfaction expressed by households in some areas, administrative authorities and local leaders expressed high levels of satisfaction with the millet distribution. For example, the village chief of Sinkarma in the Dandoli Municipality praised the aid program, indicating confidence in the amount provided. This discrepancy between household and leadership perspectives suggests a potential gap in understanding or alignment regarding community needs and the objectives of the emergency aid program. It also emphasizes the importance of integrating beneficiary feedback to bridge such gaps and ensure that the program meets the expectations of all stakeholders.

The findings underscore the critical role of adequate and equitable resource allocation in building food security resilience during periods of heightened vulnerability, such as the lean season. While households were informed that the aid was intended as a temporary measure, the reported insufficiency in some communes indicates that the distribution did not fully align with household needs. To enhance resilience and program effectiveness, humanitarian interventions should adopt a needs-based approach, considering local variations in vulnerability and food requirements. Strengthening feedback mechanisms to reconcile differing perspectives between households and local leaders can further improve the alignment of aid with beneficiary expectations and foster greater trust and cooperation in humanitarian efforts.

### 4.4. Impact of Humanitarian Aid on Food Security Needs

The findings of the study highlight the positive impact of humanitarian aid on food security in Mali, particularly through the distribution of millet and cash assistance. A substantial proportion of beneficiaries expressed satisfaction with the quality of the millet distributed, with 76% rating it as very good, 23% as good, and only 1% as average. This widespread satisfaction reflects the program's ability to meet quality expectations across most communes. Furthermore, 98% of surveyed households reported an improvement in the quality and quantity of their meals after receiving millet, enabling them to enjoy more substantial meals. However, in the commune of Kendié, 11% of households did not observe an improvement, suggesting localized challenges in achieving equitable impact. These findings emphasize the success of the millet distribution in enhancing food security and improving dietary outcomes for most beneficiaries.

The distribution of millet positively influenced food consumption patterns across the surveyed communes, with 91% of households reporting an increase in meal quantity. For many, the distribution alleviated hunger and improved overall household food security. In Dourou, the village chief testified to the program's impact, emphasizing that children no longer experienced food shortages after the distribution. However, Kendié presented a contrasting scenario, where 53% of households did not observe an improvement, primarily because they already consumed three meals per day before the aid. This disparity underscores the importance of tailoring humanitarian programs to account for pre-existing food consumption patterns. Addressing such contextual variations is essential for optimizing resource allocation and ensuring that aid reaches the most vulnerable populations.

**Figure 7:**
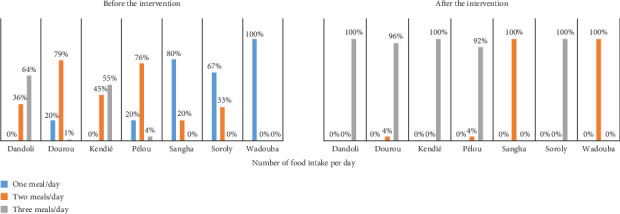
Number of meals per day before and after food distribution (field data December 2023).

The research also revealed significant disparities in food access before the millet distribution. For instance, Dandoli and Kendié had the highest rates of households receiving three meals per day (64% and 55%, respectively), while Sangha had 80% of households subsisting on only one meal. The intervention led to notable improvements, with Dandoli, Kendié, and Dourou achieving nearly universal coverage of three meals per day. Similarly, Pélou witnessed a significant shift, with most households transitioning to three meals per day, while Sangha saw an increase to two meals per day. These findings demonstrate the critical role of targeted food aid in reducing disparities and enhancing food access in vulnerable communities as indicated in Figure 7 which compares the number of food intake before and after the humanitarian assistance. Continued support and monitoring are crucial to maintaining these gains and addressing residual gaps in food security.

The success of millet distribution programs in improving food consumption patterns highlights their potential to build resilience against food insecurity. By addressing immediate dietary needs and enabling equitable access to nutrition, these interventions contribute to strengthening community resilience during periods of crisis, such as the lean season. The transition from one or two meals per day to three meals in several communes underscores the effectiveness of targeted aid in mitigating food shortages. However, the observed disparities in impact, particularly in Kendié, underscore the need for adaptive approaches that consider local contexts and pre-existing food security levels. Humanitarian agencies should integrate community feedback mechanisms and conduct regular assessments to optimize the delivery and effectiveness of aid programs, ensuring sustainable improvements in food security resilience across diverse settings.

## 5. Contribution of Humanitarian Aid to Food Security Resilience

The study evaluated the contribution of millet distribution, particularly by Caritas Switzerland, in addressing household food needs and fostering social cohesion in Mali. Findings revealed unanimous agreement among surveyed households, administrative authorities, local leaders, and cooperative officials about the positive impact of the millet distribution program on improving food security. For many households, the distributed millet effectively addressed food shortages, with 60% of respondents reporting sufficient coverage for 3 months and 40% for up to 5 months. Additionally, 100% of respondents highlighted the program's role in fostering social unity, with testimonials emphasizing how the distribution enhanced community harmony and cohesion. While these findings underline the program's immediate success in mitigating food insecurity, they also point to the need for sustainable strategies to build long-term food resilience.

### 5.1. Immediate Relief vs. Long-Term Food Resilience

The millet distribution program provided vital short-term relief, directly addressing immediate food shortages and improving household food security during the lean season. The 3–5-month coverage highlights the program's efficacy in meeting immediate needs. However, such interventions do not address the underlying structural causes of food insecurity, leaving communities vulnerable to recurring crises. This reliance on external aid underscores the necessity of transitioning from short-term emergency responses to long-term strategies that enhance food production, storage, and distribution within communities. Sustainable food security initiatives, such as investment in local agriculture, capacity-building for farmers, and improved infrastructure, are critical to reducing dependence on external aid.

### 5.2. Promoting Community Participation and Socioeconomic Development

While the distribution program successfully fostered social cohesion, leveraging community participation in planning and implementation could amplify its long-term impact. Empowering local leaders and cooperatives to play an active role in food security initiatives ensures that interventions align with community priorities and needs. Addressing socioeconomic factors contributing to food insecurity, such as poverty, limited access to education, and gender inequalities, is equally important. By integrating livelihood programs with emergency aid, humanitarian efforts can foster resilience, enabling households to generate income and improve their food security autonomously.

### 5.3. Pathways to Sustainable Food Security

The findings highlight the dual benefits of emergency food aid: immediate hunger relief and strengthened community ties. However, for lasting food resilience, a shift towards sustainable practices is essential. Developing localized agricultural systems, promoting climate-smart farming techniques, and supporting cooperatives in managing food resources can reduce vulnerability to future crises. Furthermore, long-term investments in infrastructure, such as irrigation systems and market access, are vital. To build resilience, humanitarian organizations must balance short-term food distribution with empowering communities through participatory planning, socioeconomic development, and sustainable agricultural strategies, ensuring that emergency aid serves as a bridge to enduring food security.

## 6. Conclusion and Recommendations

In conclusion, this research in the Bandiagara Region has provided valuable insights into the complex factors influencing food security, highlighting the significant role of humanitarian aid in addressing urgent food needs. The study demonstrates that targeted and context-sensitive interventions, such as millet distribution, have been effective in improving food security and meeting the immediate needs of vulnerable populations. However, it also underscores the necessity of adapting aid strategies to local contexts and ensuring equitable access to resources across different communes. The findings emphasize that while humanitarian aid has had a positive impact on food security, there is a critical need for continued expansion and refinement of these interventions to enhance their long-term effectiveness.

Based on these findings, several key recommendations are proposed to enhance the effectiveness of humanitarian aid programs. First, timely and proactive delivery of aid is essential, particularly during the lean season, to mitigate food shortages and ensure support when communities need it most. Additionally, aid strategies should be customized to the unique needs and economic activities of local communities, addressing specific challenges as seen in communes like Kendié and Soroly. Maintaining high standards in both the quality and quantity of aid is also crucial, and continuous monitoring and feedback mechanisms are needed to ensure that distribution meets beneficiaries' expectations. Furthermore, the most critical recommendation emerging from the findings is the need for a balanced approach that transitions from short-term emergency relief to long-term food resilience strategies. While the millet distribution program effectively addressed immediate food shortages, it is essential to complement such interventions with sustainable initiatives that build local agricultural capacity, improve food production and storage, and address the underlying socioeconomic factors contributing to food insecurity. This includes investing in local agriculture, promoting climate-smart farming techniques, strengthening community-based cooperatives, and enhancing infrastructure, such as irrigation systems and market access. By empowering communities through participatory planning and integrating livelihood programs with humanitarian aid, a more resilient and self-sustaining food security system can be established, reducing dependence on external assistance and ensuring long-term food security.

Future research should focus on evaluating the long-term impact of humanitarian aid on food security resilience, particularly in terms of how short-term interventions can transition into sustainable solutions. Additionally, research should explore the socioeconomic factors contributing to food insecurity, such as poverty, gender inequalities, and limited access to education, to refine strategies that address the root causes of food insecurity. Investigating the role of local agricultural innovations and climate-smart farming practices will also provide insights into how communities can adapt to environmental challenges and improve food security sustainably, ensuring that aid programs contribute to long-term resilience.

## Data Availability

All the data are presented in this paper. However, the data generated or analyzed during this study are available from the corresponding author upon reasonable request.

## References

[1] Waha K., Van Wijk M. T., Fritz S. (2018). Agricultural diversification as an important strategy for achieving food security in Africa. *Global Change Biology*.

[2] Lifland A. (2012). Starvation in the Sahel: food security in Africa. *Harvard International Review*.

[3] Ivers L. C., Cullen K. A. (2011). Food insecurity: special considerations for women. *The American Journal of Clinical Nutrition*.

[4] US Department of Agriculture (2019). Definitions of food security. https://www.ers.usda.gov/webdocs/publications/99282/err-275.pdf.

[5] United Nations (2015). Transforming our world: the 2030 agenda for sustainable development. https://sdgs.un.org/2030agenda.

[6] Food and Agriculture Organization (FAO) (2020). The state of food security and nutrition in the world. http://www.fao.org/3/ca9692en/ca9692en.pdf.

[7] FAO (2023). *The State of Food Security and Nutrition in the World 2023. Urbanization, Agrifood Systems Transformation and Healthy Diets Across the Rural–Urban Continuum*.

[8] Freeman L. (2017). Environmental change, migration, and conflict in Africa: a critical examination of the interconnections. *Journal of Environment & Development*.

[9] WFP (2024). Mali country brief. https://docs.wfp.org/api/documents/WFP-0000156968/download/?_ga=2.200149161.624808447.1716019535-313695372.1714935513.

[10] IPC (2023). Mali: acute malnutrition situation for June-October 2023 and projection for November 2023- May 2024. https://www.ipcinfo.org/ipc-country-analysis/details-map/fi/c/1156650/?iso3=MLI.

[11] CREDD Unit (2023). Strategic economic recovery framework for sustainable development 2019-2023. https://www.maliapd.org/wp-content/uploads/2019/07/Version-Finale-CREDD-2019-2023.pdf.

[12] Tounkara M., Diarra S., Maiga O., Sangare H., Diawara S. I., Ma U. S. A. I. D. (2019). *Sécurité Alimentaire au*.

[13] OCHA (2022). Mali: insecurity and declining agricultural production in certain areas will reduce people’s access to food. https://reliefweb.int/report/mali/mali-ins-curit-et-la-baisse-des-productions-agricoles-par-endroits-r-duiront-l-acc-s-des.

[14] Food Security Cluster (2023). Harmonized framework for identifying areas at risk and vulnerable populations in the Sahel and West Africa. (CH2). https://fscluster.org/sites/default/files/documents/mali_fiche_de_communication_mars2023_vf.pdf.

[15] Cabell J. F., Oelofse M. (2012). An indicator framework for assessing agroecosystem. *Ecology and Society*.

[16] Trémolières M., Walther O. J., Radil S. M. (2020). *The Geography of Conflict in North and West Africa*.

[17] Altieri M. A., Nicholls C. I., Henao A., Lana M. A. (2015). Agroecology and the design of climate change-resilient farming systems. *Agronomy for Sustainable Development*.

[18] Martin R., Sunley P. (2015). On the notion of regional economic resilience: conceptualization and explanation. *Journal of Economic Geography*.

[19] Oliver T. H., Heard M. S., Isaac N. J. B. (2015). Biodiversity and resilience of ecosystem functions. *Trends in Ecology & Evolution*.

[20] Läderach P., Ramirez-Villegas J., Prager S. D. (2021). The importance of food systems in a climate crisis for peace and security in the Sahel. *International Review of the Red Cross*.

[21] Maxwell D., McGlinchy M., Parker J., Stobaugh H. (2013). *Response Analysis & Response Choice in Food Security Crises: A Roadmap. Network Paper 73*.

